# Visual Evoked Potentials in Primary Open Angle Glaucoma

**DOI:** 10.1155/2017/9540609

**Published:** 2017-07-20

**Authors:** Mukesh Kumar Jha, Dilip Thakur, Nirmala Limbu, Badri Prasad Badhu, Bishnu Hari Paudel

**Affiliations:** ^1^Department of Physiology, Kathmandu University School of Medical Sciences, Dhulikhel, Kavre, Nepal; ^2^Department of Basic and Clinical Physiology, BP Koirala Institute of Health Sciences, Dharan, Sunsari, Nepal; ^3^Department of Ophthalmology, BP Koirala Institute of Health Sciences, Dharan, Sunsari, Nepal

## Abstract

**Background and Aims:**

Visual evoked potentials (VEPs) assess the integrity of the visual pathways from the optic nerve to the occipital cortex. Optic disc cupping and visual field loss have been associated with prolongation of latency of VEP in primary open angle glaucoma (POAG).

**Methods:**

Pattern reversal and flash VEP tests were done in consenting 20 primary open angle glaucoma eyes and 40 normal control eyes.

**Results:**

In POAG cases, the refractive error [3.51 ± 1.88 versus 1.88 ± 1.11, D, *p* = 0.001], cup-disc ratio in percent [66.00 ± 16.98 versus 28.50 ± 5.80, *p* = 0.001], intraocular pressure [19.55 ± 2.08 versus 11.65 ± 1.64, mmHg, *p* = 0.001], and automated visual field pattern standard deviation [4.13 ± 6.96 versus 1.64 ± 0.45, dB, *p* = 0.001] were significantly more than in control. The visual acuity [0.41 ± 0.29 versus 1.00 ± 0.00, *p* = 0.001], foveal visual sensitivity [25.92 ± 6.88 versus 33.48 ± 1.75, dB, *p* = 0.001], and automated visual field mean deviation [−9.63 ± 10.58 versus 0.07 ± 1.54, dB, *p* = 0.001] were significantly less in cases than in control. Among VEP variables, pattern reversal latency N145 [149.00 ± 15.75 versus 137.52 ± 15.20, ms, *p* = 0.011], flash amplitude N75 [2.18 ± .57 versus 1.47 ± .38, *μ*V, *p* = 0.001], and flash amplitude N145 [1.99 ± .39 versus 1.43 ± .38, *μ*V, *p* = 0.001] were increased in cases. The pattern reversal amplitude N75 [1.97 ± .35 versus 2.47 ± .58, *μ*V, *p* = 0.001], amplitude P100 [3.09 ± .46 versus 6.07 ± 1.44, *μ*V, *p* = 0.001], and amplitude N145 [2.21 ± .58 versus 4.45 ± 1.99, *μ*V, *p* = 0.001] were decreased in cases.

**Conclusions:**

POAG caused glaucomatous damage to optic pathway.

## 1. Introduction

The visual evoked potential (VEP) is the potential recorded from the occipital region in response to visual stimuli with a long latency response. It is the evoked response that is visible without averaging [1]. Its stimulus may be one of three types: flash, full-field pattern reversal, and half-field pattern reversal. Flash VEP is used in uncooperative patients and its latencies are more variable than the pattern reversal type. Therefore, it only tests continuity of the visual pathways. The full-field pattern reversal is used as a usual stimulus for VEP, as each eye is examined individually and especially the anterior visual pathways are evaluated well. Half-field pattern reversal that is used for localization of lesions behind the optic chiasm has reduced clinical application in modern imaging procedures [1].

Primary open angle glaucoma is generally a bilateral but not always symmetrical disease characterized by adult onset, an IOP more than 21 mm Hg at some point in the course of the disease, an open angle of normal appearance, glaucomatous optic nerve head damage, and visual field loss [2, 3]. It is a widely prevalent eye disease, characterized by an optic neuropathy, and often associated with elevated intraocular pressure, leading to characteristic visual field defects and optic nerve head damage. It is well established that damage to the ganglion cells and/or their axons produces these visual field defects. What is less clear is the extent to which the ganglion cells undergo a rapid apoptotic death as opposed to lingering in an abnormal state. If the latter holds, then it raises the possibility of neuroprotection of unhealthy retinal ganglion cells. A possible indicator of the health of the retinal ganglion cells is the latency of their response [4].

The VEP is very useful in detecting an anterior visual conduction disturbance; however, it is not specific with regard to etiology. It is very useful in evaluating visual function. It is noninvasive and has excellent temporal resolution. It can be used to assess the integrity or maturational state of the visual pathway in infants and preverbal children [5]. For recording VEP, a stimulus is presented to the subject for a selected number of times, and the cerebral responses are amplified and averaged by a computer and displayed on an oscilloscope screen or printed out on paper. It is generally elicited by the monocular stimulation of each eye, while the other is covered [5].

VEP stimulation has been used in diagnosis of glaucoma. It is used for the assessment of optic nerve diseases [6]. VEP latency can be used as a measure of early glaucomatous damage before retinal ganglion cell death occurs [4]. Hence, it is used as a marker of reversible ganglion cell damage in trials of neuroprotective agents for the treatment of glaucoma. Visual evoked potentials (VEPs) assess the integrity of the visual pathways from the optic nerve to the occipital cortex. Optic disc cupping and visual field loss have been associated with prolongation of VEP latency. Therefore, it is worth conducting a study to find the effect of glaucomatous damage on VEP latencies and amplitudes.

## 2. Objective

The objective of this paper is to study visual evoked potentials (VEPs) in primary open angle glaucoma.

## 3. Materials and Methods

This case-control study was conducted in 20 eyes diagnosed as primary open angle glaucoma (POAG) in patients aged 45 to 74 years and 40 control aged 38 to 72 years in the Electrodiagnosis Lab II (Neurophysiology Lab) of Department of Physiology, BPKIHS. The patients in BPKIHS Ophthalmology OPD meeting the inclusion criteria and controls (staffs and patient attendant) were enrolled in our study after the necessary ophthalmic assessment so as to exclude the other types of glaucoma and eye disorders. The control to case ratio was 2 : 1, that is, 40 control (*n* = 40, *m* = 24, *f* = 16) and 20 (*n* = 20, *m* = 12 and *f* = 8) POAG eyes. All the patients were on *β*-blocker (timolol) for intraocular pressure control.

### 3.1. Prerecording Procedure

Experimental setup was checked with the room temperature maintained at the thermoneutral zone (25 ± 2°C). Informed written consent was taken from each subject and they were familiarized with the laboratory conditions. The recording room was kept dark. Anthropometric variables such as age (yrs), weight (kg), height (cm), and BMI (kg/m^2^) of the subjects were recorded. Ophthalmic variables such as visual acuity, refractive error, foveal visual sensitivity, cup-disc ratio in percent, intraocular pressure, automated visual field mean deviation, and automated visual field pattern standard deviation were recorded by using VFA (Humphrey Visual Field Analyzer II 745–5935-4.1/4.1).

### 3.2. Recording Procedure and Variables Recorded

The electrodes were placed using Elefix gel after proper cleaning. According to the 10–20 international system, left occipital (LO) and right occipital (RO) electrodes were placed 5 cm left and 5 cm right, respectively, from midline occipital region. Midline-frontal (MF) electrode was placed 12 cm above the nasion, and two earthing electrodes were placed, one on vertex (*C*_*z*_) and another on the left hand. Active electrode was placed on the scalp over the visual cortex at* Oz* with the reference electrode at* Fz* and ground electrode positioned at forehead, vertex (*Cz*).

#### 3.2.1. For Pattern Reversal VEP (PR-VEP)

The subjects were asked to sit on a chair at a distance of 100 cm from the television (ONIDA 14′′ color) monitor. The monitor displayed pattern reversal checkerboard with check size of 1 degree (60 min of arc) and a small square point in the board's center was used for monocular fixation (covering one eye with a hand without pressing it) of eye at the central fixation point. This was connected to the Nihon Kohden machine (NM-420S; H636, Japan) used for recording VEP. The reversal pattern rate was set at a frequency of one per sec with 300 ms of analysis time. Averaging was done for 200 times to minimize the signal-to-noise ratio. The PR-VEP recording was done twice to check the repeatability of the VEP waves produced.

#### 3.2.2. For Flash VEP

The subjects were made to lie down in a supine position with their eyes closed. They were asked to wear a led visual stimulator (SLS-3500) which was connected to the machine. Strobe light from the stimulator was used for monocular stimulation and the flash VEP recording was done twice.

In both pattern reversal and flash VEP, peak latencies of N75, P100, and N145 and amplitudes of N75, P100, and N145 were recorded. VEP latencies were measured from the origin of recording and amplitudes were measured by drawing a baseline and measuring from baseline to peak.

### 3.3. Statistical Analysis

Data collected were first entered in the Microsoft Excel Worksheet (MS Office 2007 version). Mean and standard deviation were calculated. Depending on their distribution, Mann–Whitney* U* test was used for comparing variables between cases and controls.

## 4. Results

### 4.1. Anthropometric Variables

Among the ophthalmic variables age was significantly less in control group, whereas weight and body mass index were less and height was more in control but they were not significant. The reason for the difference in age may be correlated with onset of glaucoma in old aged person (see Table 1).

### 4.2. Ophthalmic Variables

Among the ophthalmic variables, refractive error, cup-disc ratio in percent, intraocular pressure, and automated visual field pattern standard deviation were significantly more in cases, whereas visual acuity, foveal visual sensitivity, and automated visual field mean deviation were less in cases than in control (see Table 2).

### 4.3. VEP Variables

Among the VEP variables, pattern reversal latency N145, flash amplitude N75, and flash amplitude N145 were significantly increased in the cases than in control. Pattern reversal amplitude N75, pattern reversal amplitude P100, and pattern reversal amplitude N145 were significantly decreased in the cases than in control group (see Table 3).

## 5. Discussion

This study was an attempt to compare visual evoked potentials (VEPs) in primary open angle glaucoma (POAG) patients and controls so as to find any evidence of differences in VEP latencies and amplitudes. VEP latencies are a measure of early glaucomatous damage before retinal ganglion cell death. Rodarte et al. reported an increase in multifocal VEP latencies in open angle glaucoma as compared to the control [7].

In our study, the visual acuity of the primary open angle glaucoma (POAG) patients was 0.41 ± 0.29 which is contrary to Korth [8] who found it to be below 0.7 and Bergua et al. [9] who found it to be 0.8 or better. The cup-disc ratio of the cases was 66.0 ± 16.98 which is supported by earlier study [10]. The intraocular pressure of cases in our study was 19.55 ± 2.08 supported by Korth [8] and Parisi and Massimo [10]. All the cases were on pressure reducing treatment similar to study done by Bergua et al. [9]. The foveal visual sensitivity of the subjects measured to compare the macular degeneration was found significantly less in cases. The refractive error of the cases was significantly more than control (*p* = 0.001) which meant that the corrections applied to the cases were very high and the cup-disc ratio of the cases were abnormally high which is supported by the study done by Korth [8], Bergua et al. [9], and Parisi and Massimo [10].

Pattern reversal VEP latency N145 was significantly more in cases Grippo et al. [11] (2006), Thienprasiddhi et al. [12] (2006), Shih et al. [13] (1991), Bengtsson and Choong [14] (2002), and Ambrosio et al. [15] (2003). The pattern reversal latency N75 and P100 of cases was longer than the control but was not significant. All the pattern reversal VEP amplitudes (N75, N100, and N145) were significantly lesser in cases, similar to the studies done by Grippo et al. [11] (2006), Bergua et al. [9] (2004), Parisi and Massimo [10] (1992), Thienprasiddhi et al. [12] (2006), Shih et al. [13] (1991), Bengtsson and Choong [14] (2002), and Ambrosio et al. [15] (2003).

Surprisingly, the flash VEP amplitude (N75 and N145) was significantly higher in cases. The flash VEP latencies (N75, P100, and N145) were longer in cases but were not significant. Similarly, the flash VEP amplitude P100 was lesser in cases but was also not significant.

When pattern reversal VEP was compared with the flash VEP variables, P100 and N145 latency pattern reversal latency was significantly less. However, the pattern reversal N75 latency was also less but was not significant. The pattern reversal amplitudes (N75, P100, and N145) were significantly higher as compared to the flash VEP. This indicates that pattern reversal VEP is more reliable than flash VEP in clinical diagnosis (Aminoff) [4].

A positive correlation was seen between pattern reversal VEP latencies N75 and P100 (*r* = 0.559, *p* = 0.001); N75 and N145 (*r* = 0.586, *p* = 0.001); and P100 and N145 (*r* = 0.536, *p* = 0.001). The pattern reversal VEP latency P100 showed positive correlation with pattern reversal amplitude P100 (*r* = 0.464, *p* = 0.003). Similarly, the flash VEP latencies N75 and P100 (*r* = 0.425, *p* = 0.006); P100 and N145 (*r* = 0.415, *p* = 0.008); N75 and N145 (*r* = 0.364, *p* = 0.021); and flash VEP amplitudes N75 and P100 (*r* = 0.412, *p* = 0.008) showed positive correlation.

The average age of the POAG cases was more than the control. The control enrolled in the study had normal automated visual field (AVF). A certain degree of peripheral vision loss in each individual with the advancement of age is natural. So, exact age matched normal could not be taken. Accordingly in a study by Aminoff [4], controls were on an average 10 years younger than the patient. However, the effects of age were relatively smaller (1.3 ms/decade) for the monocular latency measures and very small (0.1 ms/decade) for the interocular latency measures. This suggests that the relatively smaller increase in latency that was found for the patients might be slightly smaller if age was taken into consideration [4]. Also the report of flash VEP was not so conclusive to compare with PR-VEP. These were the limitations of the study. Nevertheless, this study has created a preliminary normative data for clinical reporting purpose and it can be utilized as a background for further studies in the related field. In this way, this study holds a big strength.

## 6. Conclusion

It is concluded that POAG caused deterioration in all the ophthalmic variables measured with increase in latency. The amplitude is different between pattern reversal and flash methods.

### 6.1. Future Directions

Further study can be done to make flash VEP more conclusive and useful for clinical interpretation. Also, the VEP of the subjects of different age group can be studied to evaluate the effect of age on VEP.

## Figures and Tables

**Table 1:** Anthropometric variables of the cases and control.

Variables	Groups		*p* value
	Cases (*n* = 10) (mean ± SD)	Control (*n* = 20) (mean ± SD)	
Age (years)	63.3 ± 9.02	48.3 ± 8.65	0.001
Weight (kg)	62.3 ± 8.87	61.6 ± 6.38	NS
Height (cm)	163.3 ± 9.02	163.9 ± 5.63	NS
Body mass index (kg/m^2^)	23.2 ± 1.82	22.8 ± 1.47	NS

**Table 2:** Comparison of ophthalmic variables between cases and control.

Variables	Groups		*p* value
	Cases (*n* = 20) (mean ± SD)	Control (*n* = 40) (mean ± SD)	
VA	0.41 ± 0.29	1.00 ± 0.00	0.001
RE (D)	3.51 ± 1.88	1.88 ± 1.11	0.001
FVS (dB)	25.92 ± 6.88	33.48 ± 1.75	0.001
CDRP	66.00 ± 16.98	28.50 ± 5.80	0.001
IOP (mmHg)	19.55 ± 2.08	11.65 ± 1.64	0.001
AVFMD (dB)	−9.63 ± 10.58	0.07 ± 1.54	0.001
AVFPSD (dB)	4.13 ± 6.96	1.64 ± 0.45	0.001

VA = visual acuity in decimal system; RE = refractive error; FVS = foveal visual sensitivity; CDRP = cup-disc ratio in percent; IOP = intraocular pressure; AVFMD = automated visual field mean deviation; AVFPSD = automated visual field pattern standard deviation.

**Table 3:** VEP variables of POAG cases and control.

Variables	Groups		*p* value
	Cases (*n* = 20) (mean ± SD)	Control (*n* = 40) (mean ± SD)	
PL75 (ms)	68.53 ± 12.34	67.30 ± 5.09	NS
PL100 (ms)	103.21 ± 10.82	98.25 ± 4.05	NS
PL145 (ms)	149.00 ± 15.75	137.52 ± 15.20	0.011
PA75 (*µ*V)	1.97 ± 0.35	2.47 ± 0.58	0.001
PA100 (*µ*V)	3.09 ± 0.46	6.07 ± 1.44	0.001
PA145 (*µ*V)	2.21 ± 0.58	4.45 ± 1.99	0.001
FL75 (ms)	75.53 ± 15.55	66.85 ± 6.13	NS
FL100 (ms)	109.71 ± 18.38	101.90 ± 7.02	NS
FL145 (ms)	149.47 ± 19.74	146.62 ± 13.22	NS
FA75 (*µ*V)	2.18 ± 0.57	1.47 ± 0.38	0.001
FA100 (*µ*V)	2.50 ± 0.81	2.78 ± 0.42	NS
FA145 (*µ*V)	1.99 ± 0.39	1.43 ± 0.38	0.001

PL75 = pattern reversal latency; PL100 = pattern reversal latency; PL145 = pattern reversal latency; PA75 = pattern reversal amplitude; PA100 = pattern reversal amplitude; PA145 = pattern reversal amplitude; FL75 = flash latency; FL100 = flash latency; FL145 = flash latency; FA75 = flash amplitude; FA100 = flash amplitude; FA145 = flash amplitude.

## References

[1] Misulis K. E., Head T. C. (pp. 201–209, 2003). *Essentials of Clinical Neurophysiology*.

[2] Shields M. B. (pp. 153–176, 1998). *Textbook of Glaucoma*.

[3] Ricorda-Eva P., Whitcher J. P. (2007). *Vaughan & Asbury’s General Ophthalmology*.

[4] Aminoff M. J. (pp. 421–449, 1999). *Electrodiagnosis in Clinical Neurology*.

[5] Johnson C. A., Samuels S. J. (1997). Screening for glaucomatous visual field loss with frequency-doubling perimetry. *Investigative Ophthalmology & Visual Science*.

[6] Momose K., Kiyosawa M., Nemoto N., Mori H., Mochizuki M., Yu J. J.-H. (2004). PRBS-determined temporal frequency characteristics of VEP in glaucoma. *Documenta Ophthalmologica*.

[7] Rodarte C., Hood D. C., Yang E. B. (2006). The effects of glaucoma on the latency of the multifocal visual evoked potential. *British Journal of Ophthalmology*.

[8] Korth M. (1997). The value of electrophysiological testing in glaucomatous diseases. *Journal of Glaucoma*.

[9] Bergua A., Horn F. K., Martus P., Jünemann A. M., Korth M. (2004). Stereoscopic visual evoked potentials in normal subjects and patients with open-angle glaucomas. *Graefe's Archive for Clinical and Experimental Ophthalmology*.

[10] Parisi V., Massimo G. B. (1992). Visual evoked potentials after photostress in patients with primary open angle glaucoma and ocular hypertension. *Investigative Ophthalmology & Visual Science*.

[11] Grippo T. M., Hood D. C., Kanadani F. N. (2006). A comparison between multifocal and conventional VEP latency changes secondary to glaucomatous damage. *Investigative Ophthalmology and Visual Science*.

[12] Thienprasiddhi P., Greenstein V. C., Chu D. H. (2006). Detecting early functional damage in glaucoma suspect and ocular hypertensive patients with the multifocal VEP technique. *Journal of Glaucoma*.

[13] Shih Y.-H., Huang Z.-J., Chang C.-E. (1991). Color pattern-reversal visual evoked potential in eyes with ocular hypertension and primary open-angle glaucoma. *Documenta Ophthalmologica*.

[14] Bengtsson B., Choong Y. F. (2002). Evaluation of VEP perimetry in normal subjects and glaucoma patients. *Acta Ophthalmologica Scandinavica*.

[15] Ambrosio G., Ferrara G., Vitale R., De Marco R. (2003). Visual evoked potentials in patients with Graves' ophthalmopathy complicated by ocular hypertension and suspect glaucoma or dysthyroid optic neuropathy. *Documenta Ophthalmologica*.

